# Contrast-enhanced ultrasound features of malignant focal liver masses in dogs

**DOI:** 10.1038/s41598-020-63220-3

**Published:** 2020-04-08

**Authors:** Silvia Burti, Alessandro Zotti, Giuseppe Rubini, Riccardo Orlandi, Paolo Bargellini, Federico Bonsembiante, Tommaso Banzato

**Affiliations:** 1 Department of Animal Medicine, Productions and Health, University of Padua, Viale dell’Università 16, Legnaro, Italy; 2 ULTRAVET, Via E. Fermi 59, San Giovanni in Persiceto, Bologna, Italy; 3 Tyrus Veterinary Clinic, Via A. Bartocci 1/G, Terni, Italy; 4 Department of Comparative Biomedicine and Food Science, University of Padua, Viale dell’Università 16, Legnaro, Italy

**Keywords:** Medical imaging, Ultrasonography

## Abstract

A total of 185 cases (150 retrospectively and 35 prospectively) of malignant liver masses were collected. In the retrospectively collected cases hyperenhancement during wash-in was the most common feature in HCCs but there was a high percentage of cases showing no enhancement or hypo/isoenhancement. ICCs displayed a large variety of contrast enhancement patterns and, although statically significant differences between ICCs and HCCs were evident, no clear distinction between these two pathologies was possible based only on their CEUS appearance. Sarcomas displayed all the possible degrees of wash-in enhancement with non-enhancing being the most common appearance. Metastases displayed all the possible contrast-enhancement patterns, with the most common being hyperenhancement in the wash-in phase followed by hypoenhancement in the wash-out phase. A decision tree was developed based on the features of the retrospectively selected cases. Based on the developed decision tree 27/35 prospectively collected cases were correctly classified. Even if some significant differences among groups were evident, all the histotypes displayed all the possible patterns of contrast enhancement, and, therefore, the differentiation of liver masses in dogs based only on their CEUS features is not feasible and, therefore, cytology or histopathology is required.

## Introduction

Primary hepatobiliary tumours are uncommon in dogs, accounting for less than 1.5% of all canine neoplasms. Metastases from extra-hepatic tumours, particularly from splenic, pancreatic and gastrointestinal tract neoplasms, are 2.5 times more frequent^1^. In dogs, as in humans, primary liver tumours are classified according to their histological origin. Tumours originating from hepatocytes are classified as hepatocellular adenomas or carcinomas (HCC), tumours arising from the bile duct epithelium are classified as biliary adenoma or cholangiocarcinoma (ICC); sarcomas arise from stromal cells, and neuroendocrine carcinomas originate from neuroendocrine cells^2^. HCCs are the most common primary liver neoplasia in dogs, accounting for about 50% of cases, followed by ICCs, which account for about 22 to 41% of cases; neuroendocrine carcinomas, and sarcomas have rarely been reported^1^.

B-mode ultrasound (US) is the most commonly used diagnostic imaging technique for liver evaluation, both in human and veterinary medicine. The identification of focal liver lesions with US is, in most cases, straightforward^3^ but, unfortunately, US alone does not provide useful features to determine their histological origin^4,5^. Furthermore, some lesions are impossible to detect by means of US due to their similarity to the liver parenchyma. Therefore, additional examinations, such as magnetic resonance imaging, computed tomography, contrast-enhanced ultrasound (CEUS), along with cytopathological or histological examination are required to determine the histotype of each lesion. Treatment options for canine hepatic neoplasms depend on the location, the distribution and the histotype of the lesion^1^. The scope to accurately predict histotype and lesion spread plays a fundamental role in treating the oncological patient.

In the last decade some research papers regarding the use of CEUS in the evaluation of canine^6–8^ and feline^9^ focal liver lesions have been published but, compared to the large number of articles available on this topic in human medicine, the veterinary literature is scarce and based on a small number of cases. Moreover, there are no large studies that describe CEUS features of malignant liver lesions in order to predict tumor histotype, and only few cases are presented on this topic in the veterinary field^6,8^. Therefore, this study has three aims: (1) to describe the CEUS features of malignant canine liver lesions, based on a relatively large number of retrospectively collected cases; (2) to compare such features with those reported in the literature; (3) to test the accuracy of CEUS in the prediction of tumour histotype on prospectively collected cases.

## Results

### Retrospective collection of cases

A total of 166 dogs of different breeds were collected, 90 females and 76 males, with a mean age of 11.3 years (standard deviation ± 2.9). Sixteen dogs were excluded from the study: 9 because the cytological diagnosis was uncertain and a benign condition could not be excluded, and 7 were diagnosed with diffuse malignancy (lymphoma). Based on the inclusion criteria, 150 dogs were included: 68 had a final diagnosis of HCC, 26 of ICC, 46 of sarcoma (24 hemangiosarcomas,11 metastases of sarcoma, 11 lesions were classified as undifferentiated sarcoma due to marked cell atypia), and 10 cases were classified as metastasis from primary carcinoma (1 apocrine gland adenocarcinoma of the anal sac, 2 mammary carcinomas, 2 pancreatic carcinomas, 1 oral melanoma, 4 lesions from undifferentiated primary carcinoma due to marked atypia of the cells).

### Analysis of B-mode examinations

Summary statistics of B-mode ultrasonographic qualitative features of liver masses with cytopathological classification are reported in Table 1. Only the distribution of the lesions was statistically different among the cytopathological groups (χ^2^ = 20.115; p < 0.001), with HCCs being mainly focal (45/68), and sarcomas being mostly diffused (35/46). ICCs and metastases showed no characteristic diffusion pattern. No statistically significant differences were also evident for the echogenicity (χ^2^ = 10.114; p = 0.341) and in the aspect (χ^2^ = 1.207; p = 0.752) of the lesions in relation to the histotype.

**Table 1:** Number of cases showing qualitative features at B-mode ultrasound.

	Isoechoic	Echogenicity		Mixed	Aspect		Distribution	
		Hypoechoic	Hyperechoic		Solid	Cystic	Focal	Diffuse
**Cytological diagnosis**								
ICC	1	6	7	11	17	8	11	14
HCC	1	15	26	26	44	24	45	23
Sarcoma	0	7	15	24	31	15	11	35
Metastasis	1	0	5	4	5	5	4	6
P-Value	0.34	0.75	<0.001					

### Analysis of CEUS examinations

The degree of wash-in enhancement was statistically different between cytopathological groups (χ^2^ = 58.508; p < 0.002). Post-hoc tests revealed significant differences between ICCs and HCCs (p = 0.002), HCCs and sarcomas (p < 0.001), HCCs and metastases (p = 0.027), and ICCs and sarcomas (p = 0.003). A distinctive characteristic of HCCs was hyperenhancement during the wash-in phase (50/68), whereas ICCs displayed all degrees of wash-in enhancement. Moreover, the sarcomas showed all the possible enhancement degrees, with no enhancement (20/46) being the most common feature. Metastases showed no distinctive wash-in enhancement features. Only the wash-in enhancement-degree, and the evaluation of the margin characteristics was considered in the statistical analysis of not-enhancing masses (1 ICC, 3 HCCs, 20 sarcomas, and 3 metastases).

The distribution of contrast medium during wash-in (χ^2^ = 13.720; p = 0.033) showed limited statistically significant differences between groups, while pairwise comparisons showed significant differences only between HCCs and sarcomas (p = 0.020). Indeed, ICCs and HCCs in most cases showed diffuse contrast-medium distribution (15/26 and 50/68 respectively), while sarcomas and metastases showed equally peripheral and diffuse distribution. No significant differences in the homogeneity of contrast-medium distribution were evident during wash-in (χ^2^ = 5.102; p = 0.164). Complete results of wash-in phase are reported in Table 2.

**Table 2:** Number of cases, classified based on cytological examination, showing each contrast-enhanced qualitative feature during wash-in. The P-value of the Chi-squared test is also reported.

	Enhancement degree				Homogeneity		Distribution		
	Isoenhancing	Hypoenhancing	Hyperenhancing	Not enhancing	Homogenous	Inhomogeneous	Central	Peripheral	Diffuse
**Cytological diagnosis**									
ICC	4	12	8	1	7	17	1	9	14
HCC	8	7	50	3	13	52	4	11	50
Sarcoma	3	14	9	20	8	18	0	13	13
Metastasis	1	2	4	3	4	3	1	3	3
**P-Value**	<0.001*				0.164		0.033*		

Number of cases showing qualitative contrast-enhancement features evaluated during wash-in phase, as classified by cytopathology.

* indicates statistical significance.

The degree of wash-out enhancement (χ^2^ = 10.452; p = 0.015) showed statistically significant differences, but pairwise comparisons revealed no differences between groups. A distinctive characteristic for ICCs and sarcomas is having no wash-out (18/26 and 21/46 respectively), while HCCs showed both “no wash-out” and hypoenhancing degree during wash-out phase. No significant differences in the wash-out pattern of the contrast-medium distribution (χ^2^ = 13.982; p = 0.123) were evident. Complete results about wash-out phase are reported in Table 3.

**Table 3:** Number of cases, classified based on cytological examination, showing each contrast-enhanced qualitative feature during wash-out. The P-value of the Chi-squared test is also reported.

	Enhacement degree		Homogeneity		Pattern		
	Hypoenhancing	No wash out	Homogeneous	Inhomogeneous	Centripetal	Centrifugal	Diffuse
**Cytological diagnosis**							
ICC	7	17	1	6	0	0	7
HCC	32	33	1	31	2	1	29
Sarcoma	5	21	2	3	0	1	4
Metastasis	5	2	2	3	0	0	5
**P-Value**	0.015*		0.001*		0.123		

Number of cases showing the qualitative contrast-enhancement features evaluated during the wash-out phase, as classified by cytopathology.

* indicates statistical significance.

**Figure 1:**
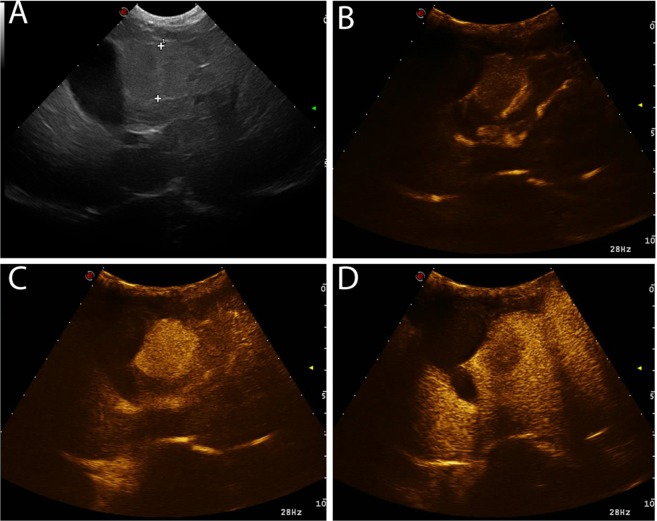
Example of an HCC showing a hyperenhancing wash-in and a hypoenhancing wash-out. The images represent the different time points of the CEUS examination evaluated in the study: (**a**) B-mode image of the lesion; (**b**) TTE; (**c**) TTP; (**d**) wash-out.

**Figure 2:**
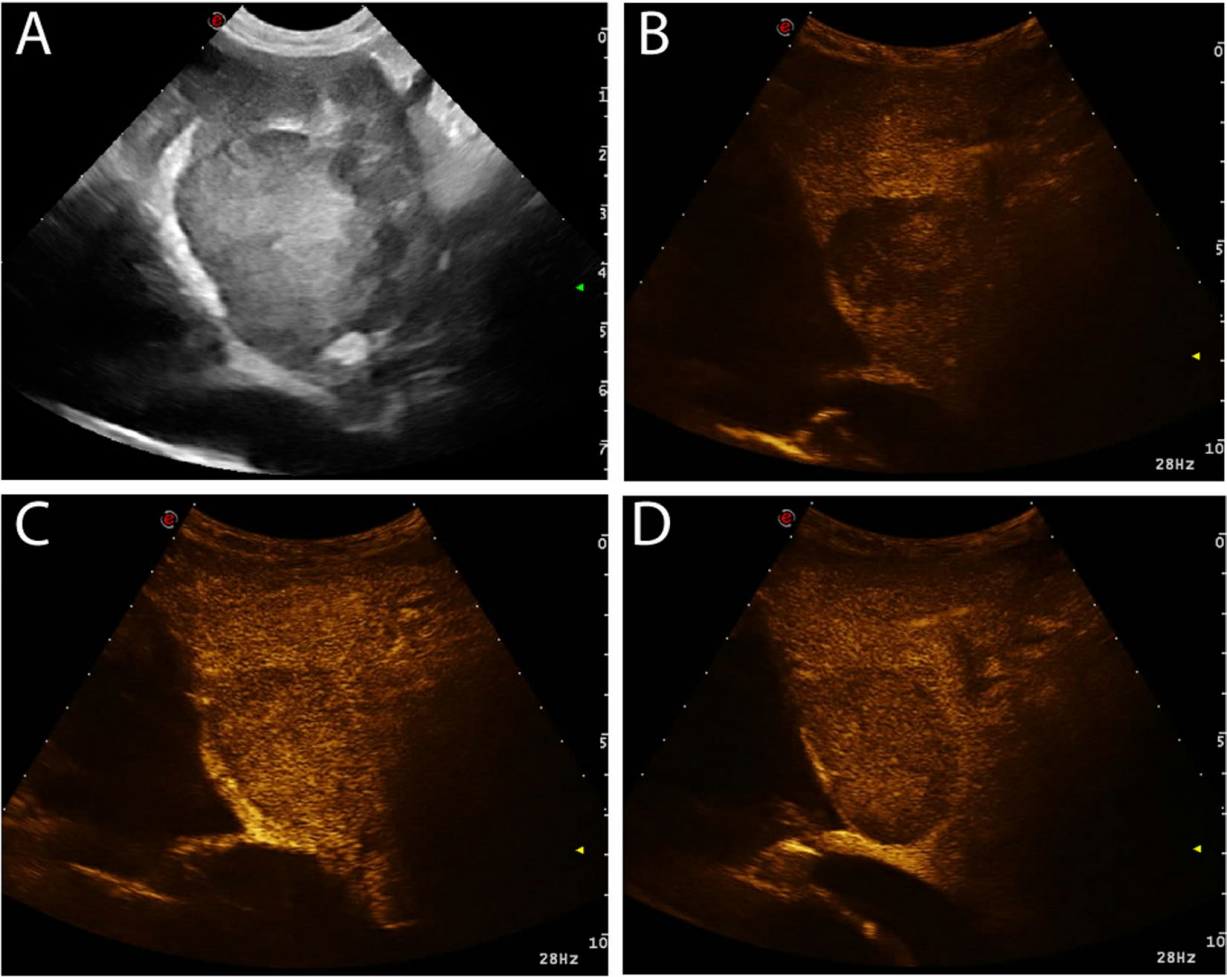
Example of a ICC showing hypoenhancing wash-in and hypoenhancing wash-out and, therefore, this lesion was classified as having “no wash-out”. The images represent the different time points of the CEUS examination evaluated in the study: (**a**) B-mode image of the lesion; (**b**) TTE; (**c**) TTP; (**d**) wash-out.

**Figure 3:**
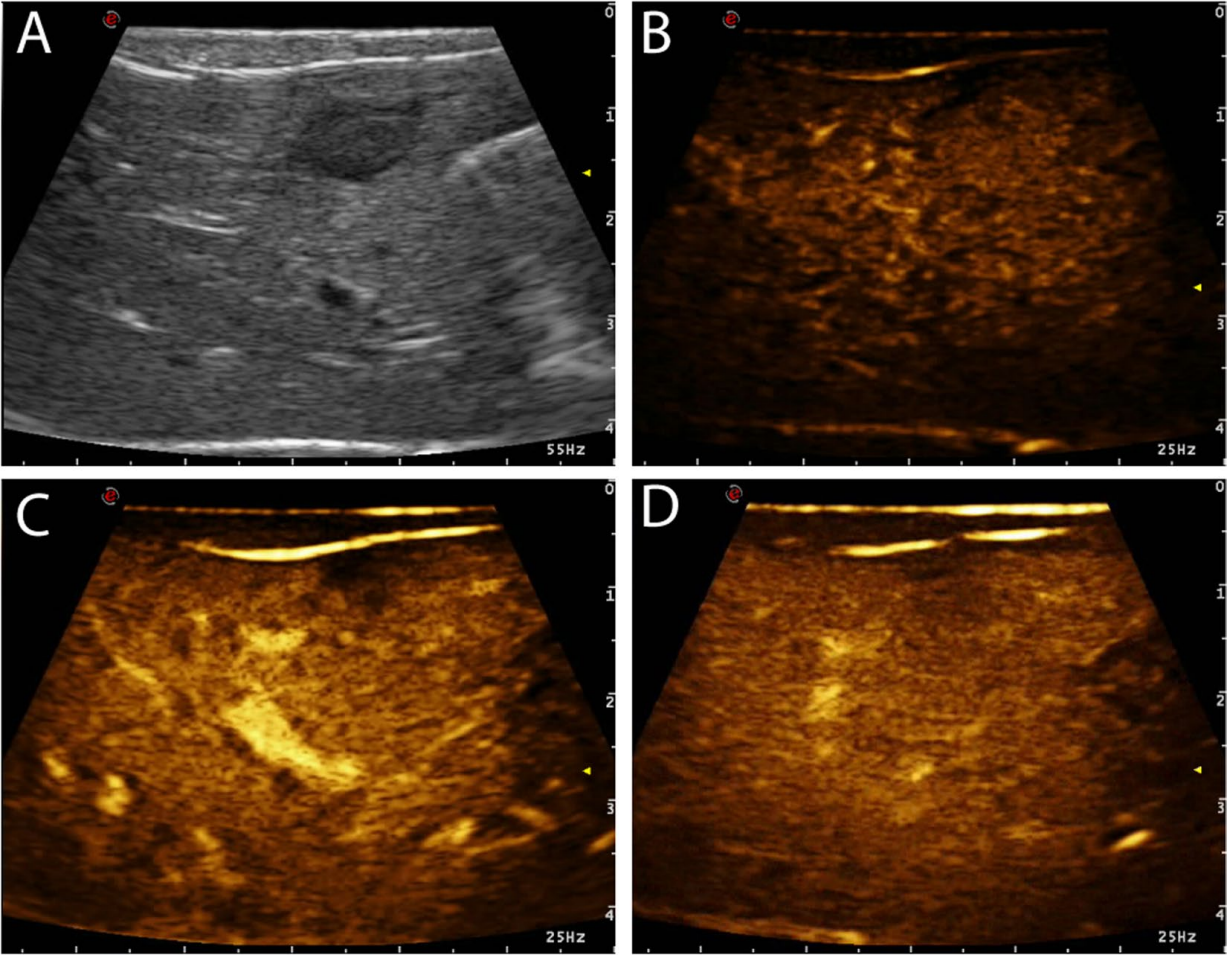
Example of ICC characterised by isoenhancing wash-in and isoenhancing wash-out and, therefore, this lesion was classified as having “no wash-out”. The images represent the different time points of the CEUS examination that were evaluated in the study: (**a**) B-mode image of the lesion; (**b**) TTE; (**c**) TTP; (**d**) wash-out.

**Figure 4:**
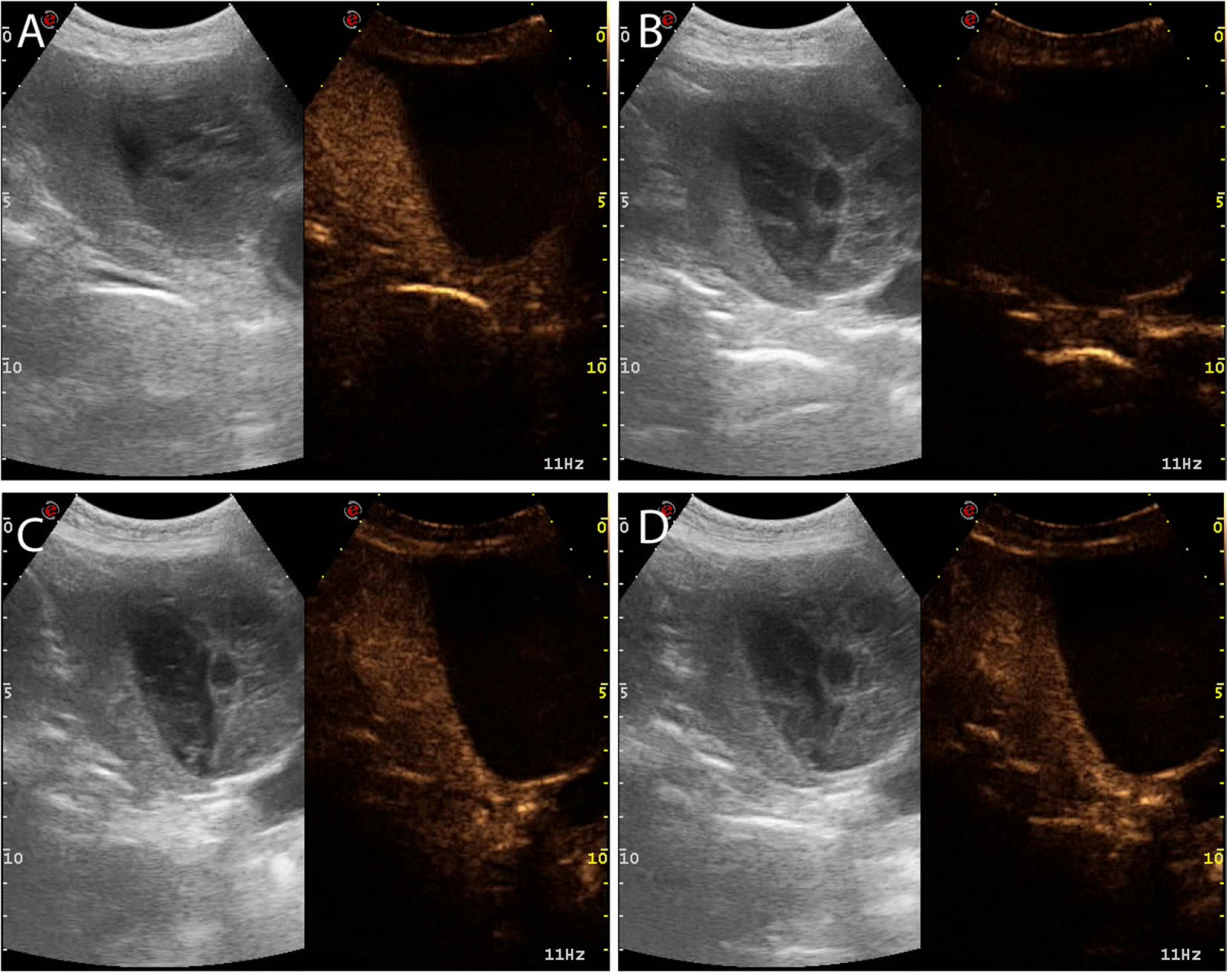
Example of an HCC showing not-enhancement during the entire CEUS examination. The images represent four different time points.

**Figure 5:**
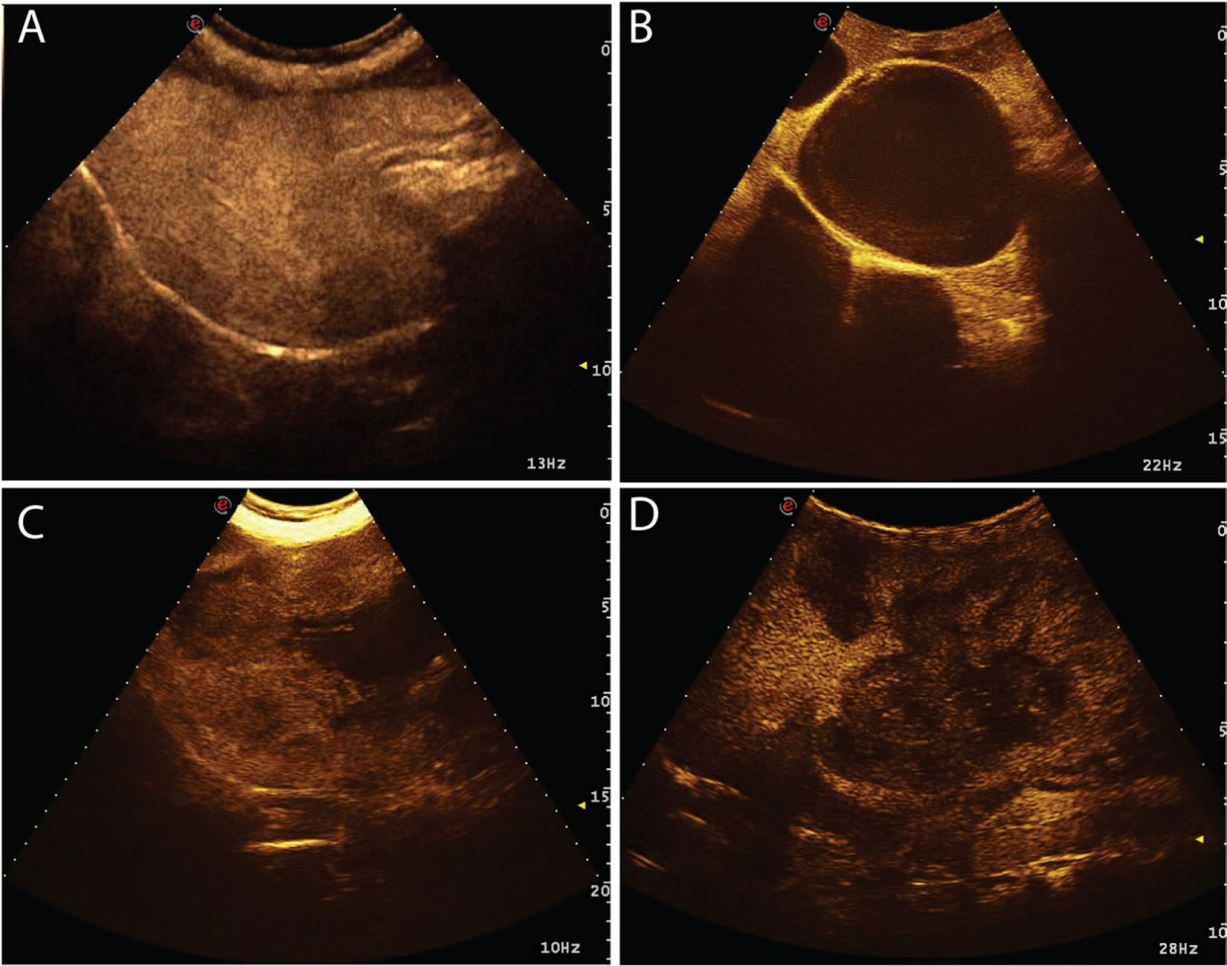
Example of the different margin characteristics that were evaluated for each lesion during the entire CEUS examination: (**a**) HCC showing unclear and regular margins; (**b**) sarcoma showing clear and regular margins; (**c**) ICC showing unclear and irregular margins; (**d**) metastasis showing clear and irregular margins.

**Figure 6:**
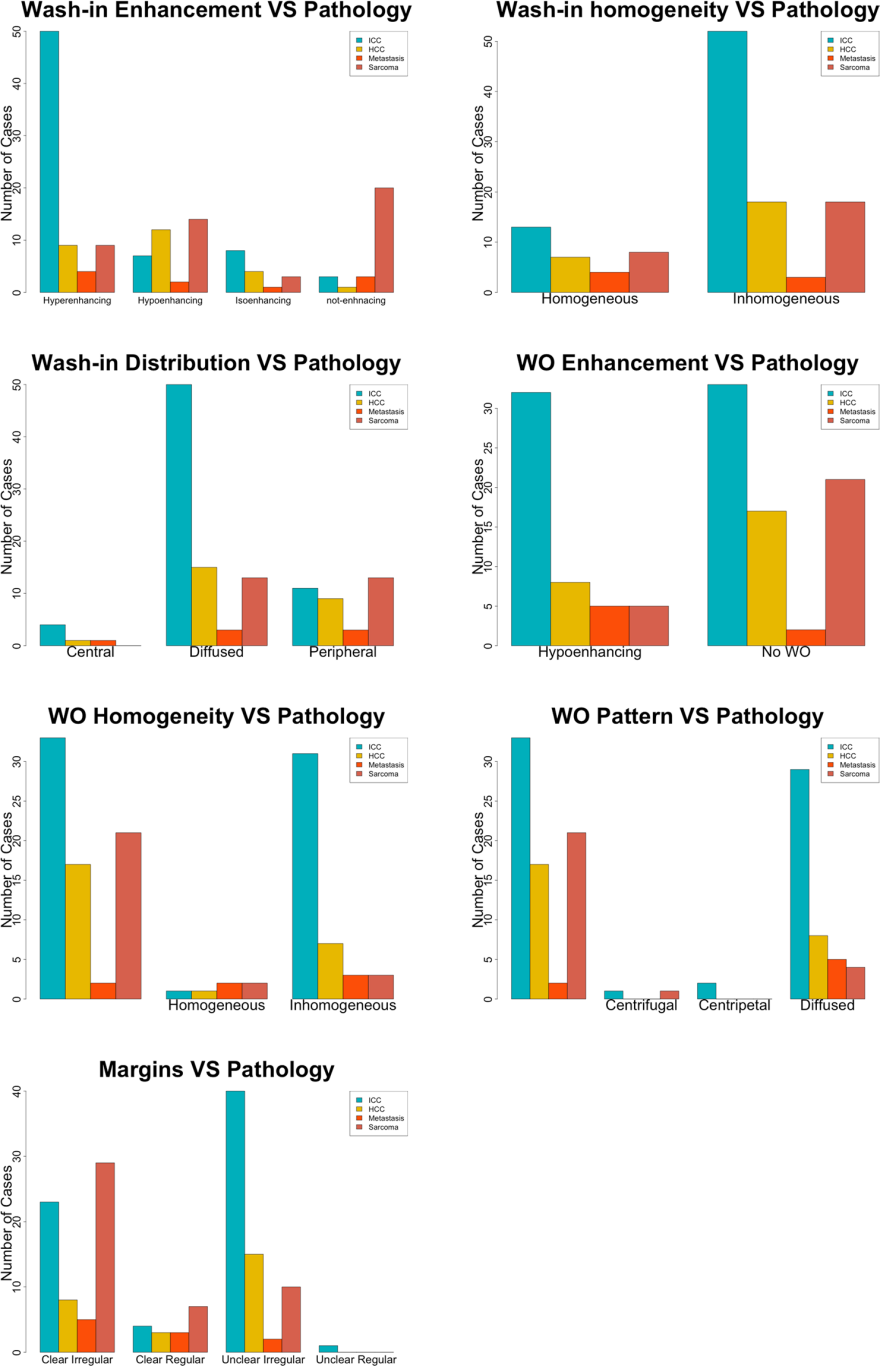
Bar plots of the distribution of the qualitative features.

The characteristics of the margins showed statistically significant differences between cytopathological groups (χ^2^ = 24.084; p = 0.005). Margin characteristics resulted significantly different between ICCs and sarcomas (p = 0.025), and between HCCs and sarcomas (p = 0.001). Indeed, ICCs had mainly unclear and irregular margins (15/26), whereas sarcomas showed mainly clear and irregular margins (29/46). HCCs showed both clear and irregular (23/68), and unclear and irregular (40/68) margins. Complete results of the margins are reported in Table 4. The most common qualitative features of each histotypes, as a result of the statistical analysis, considered are reported in Table 5. Representative cases of the different enhancement patterns are reported in Figs. 1–4. Representative cases of the characteristics of the margins are reported in Fig. 5. Bar plots of the distribution of the qualitative features are reported in Fig. 6.

**Table 4:** Characteristics of the margins of the lesions as classified by cytopathological examination. The P-value of the Chi-squared test is also reported.

	Margins			
	Clear and regular	Clear and irregular	Unclear and regular	Unclear and irregular
**Cytological diagnosis**				
ICC	3	8	0	14
HCC	4	23	1	40
Sarcoma	7	29	0	10
Metastasis	3	5	0	2
**P-Value**	0.005*			

Number of cases showing different lesion margins during the overall CEUS examination.

* indicates statistical significance.

**Table 5:** Distinctive characteristics for each tumor among the CEUS qualitative features evaluated, during wash-in and wash-out phases.

	Wash-in			Wash-out			Margins
	Enhancement degree	Homogeneity	Distribution	Enhancement degree	Homogeneity	Pattern	
**Cytological diagnosis**							
ICC	Hypoenhancing	Inhomogeneous	Diffuse	No wash-out	Inhomogeneous	Diffuse	Unclear Irregular
HCC	Hyperenhancing	Inhomogeneous	Diffuse	No wash-out	Inhomogeneous	Diffuse	Unclear Irregular
Sarcoma	Not-Enhancing	Inhomogeneous	Peripheral/Diffuse	No wash-out	Inhomogeneous	Diffuse	Clear Irregular
Metastasis	Hyperenhancing	Homogeneous	Peripheral/Diffuse	Hypoenhancing	Inhomogeneous	Diffuse	Clear Irregular

**Figure 7:**
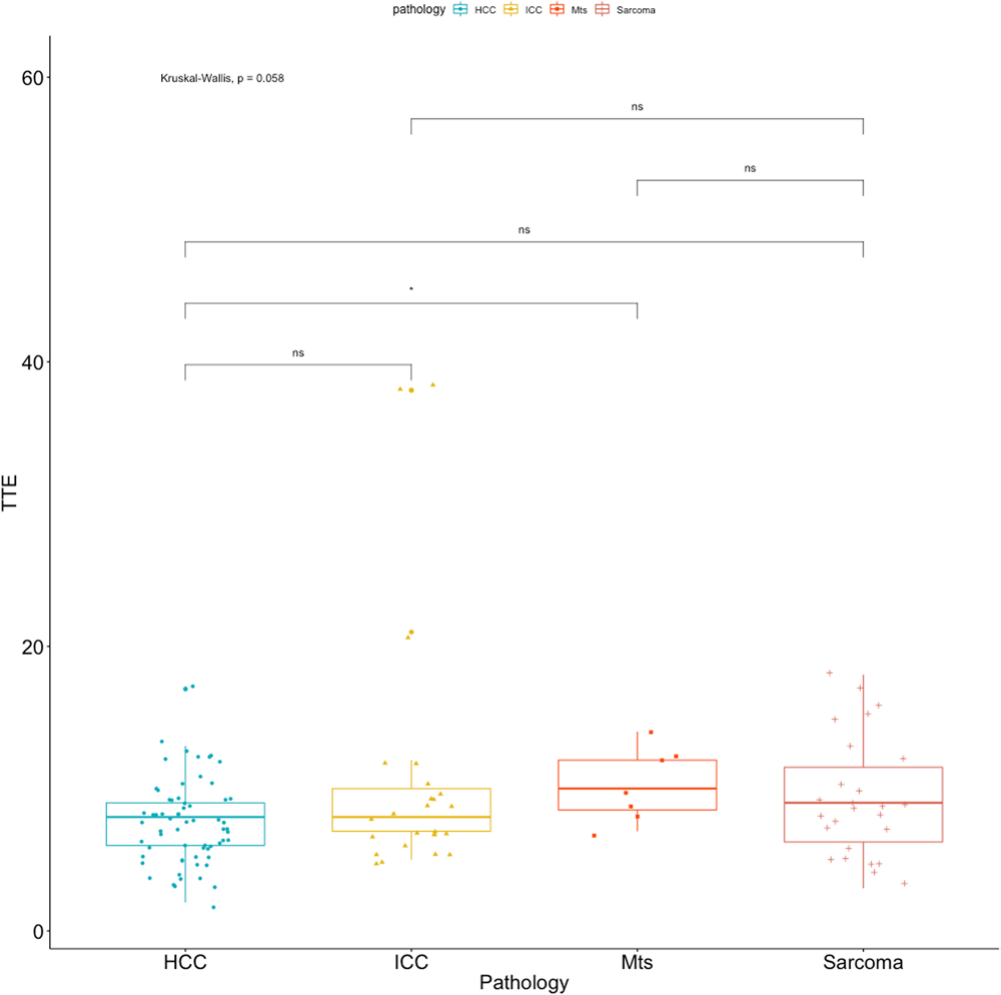
Box plot of the TTE, along with the results of the pairwise comparisons, as classified by cytopathology.

**Figure 8:**
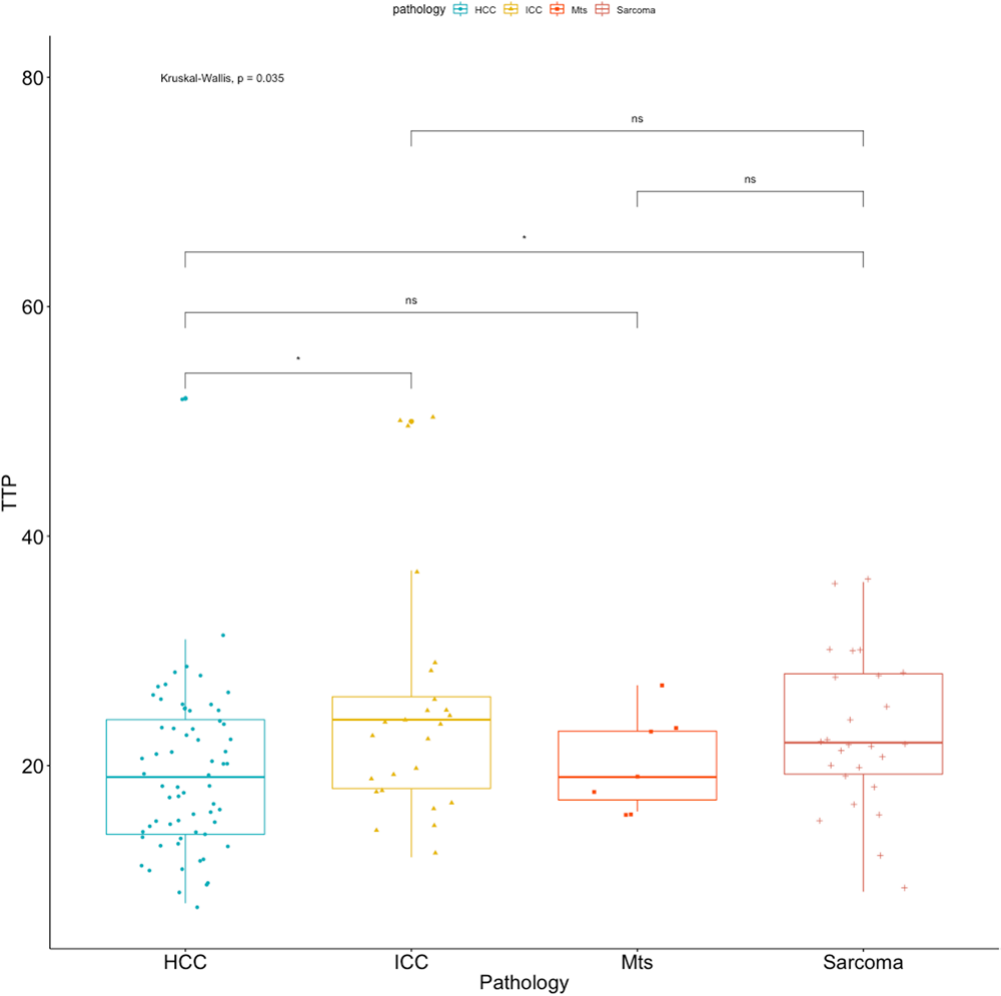
Box plot of the TTP, along with the results of the pairwise comparisons, as classified by cytopathology.

**Figure 9:**
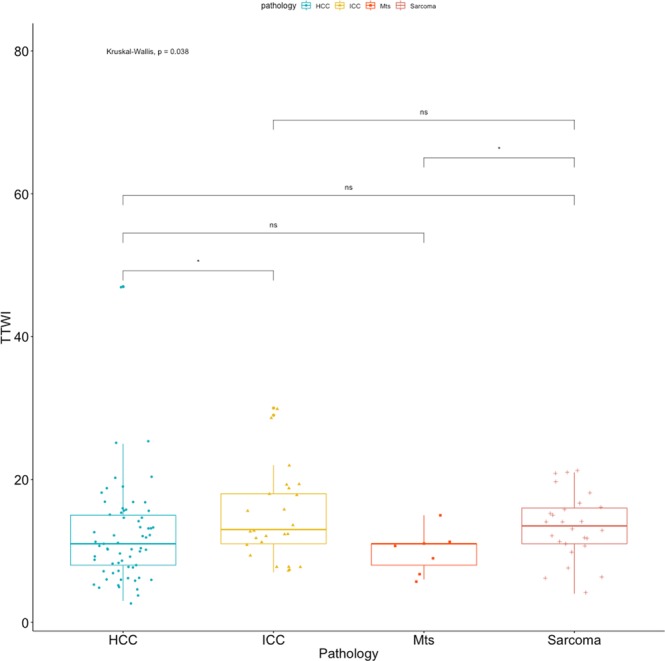
Box plot of the TTWI, along with the results of the pairwise comparisons, as classified by cytopathology.

All the quantitative features were not-normally distributed and, therefore, differences between groups were calculated using the Kruskal-Wallis test. Statistically significant differences between cytopathological groups were evident for the TTP (F = 8.567; p = 0.035), the TTWI (F = 8.367; p = 0.038) but not for the TTE (F = 7.405; p = 0.057). Summary statistics of the quantitative features for the different cytopathological groups, excluding not-enhancing lesions, are reported in Table 6. Box plots of the TTE, the TTP and the TTWI, along with the results of the pairwise comparisons, are reported in Figs. 7–9 respectively. Example of time-intensity curves used to calculate TTE, TTP and TTWI can be found as Supplementary Fig. S1.

**Table 6:** Contrast-enhanced quantitative features of the lesions classified on the basis of cytological examinations. The P-value of the Kruskal-Wallis test is also reported.

	TTE	TTP	TTWI (TTP - TTE)
**Cytological diagnosis**			
ICC	9.50 (5–38)	24.12 (12–50)	14.62 (7–30)
HCC	7.55 (2–17)	19.46 (8–52)	11.91 (3–47)
Sarcoma	9.31 (3–18)	22.81 (9–36)	13.50 (4–21)
Metastasis	10.29 (7–14)	20.29 (16–27)	10.00 (6–15)
**P-Value**	0.057	0.035	0.038

Values are reported as mean with limits of the overall range.

### Prospective cases analysis

**Figure 10:**
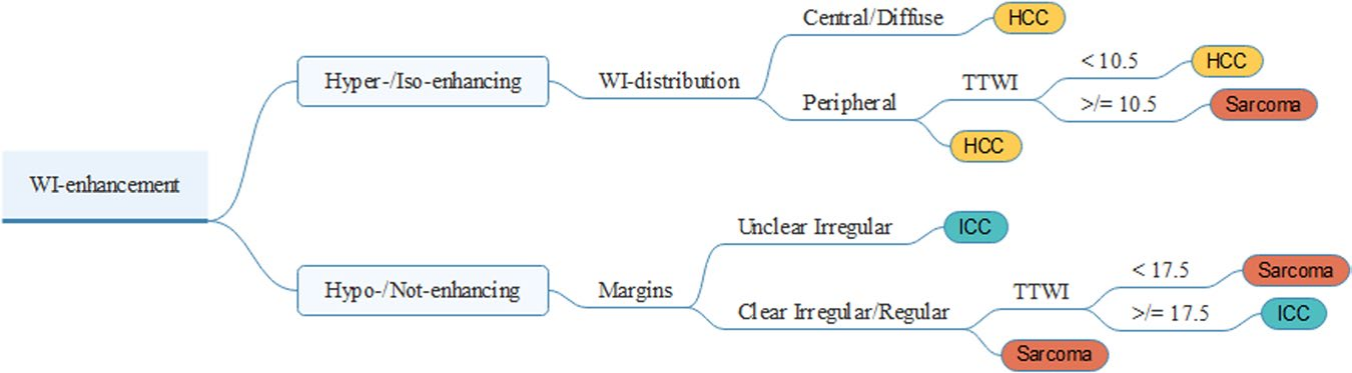
The developed decision tree based on the CEUS features of the retrospectively collected cases. To classify the cases the starting point is the WI-enhancement, thereafter the different branches are followed until the end for final prediction.

Thirty-five patients were collected in the prospective phase of the study (3 ICCs, 28 HCCs, 3 sarcomas, and 1 metastasis), 24 females and 11 males, with mean age of 12.3 years (standard deviation ± 3.2). 3 patients had a final diagnosis of ICC, 28 of HCC, 3 of sarcoma, and 1 of metastasis from cutaneous melanoma. Complete results of the analysis of the B-mode and CEUS examinations can be forund as Supplementary Table S1. Due to their reduced statistical significance B-mode features were not included in the development of the decision tree. The metastasis from cutaneous melanoma was excluded from the analysis. An overall accuracy of 0.79 was obtained using the decision tree to classify the new cases based on their CEUS features. The decision tree had a very high precision for HCC and a very high recall for sarcoma. Indeed, all the cases classified as HCCs were actually HCCs, and all the sarcoma cases were correctly classified. On the other hand, 6/28 (21.44%) HCCs were incorrectly classified based on their CEUS features. The decision tree is reported in Fig. 10. Complete results of the analysis are reported in Table 7.

**Table 7:** Detailed results of the classification of the prospective cases using the developed decision tree.

	Balanced accuracy	Precision	Recall
**Cytological diagnosis**			
ICC	0.78	0.4	0.67
HCC	0.89	1	0.78
Sarcoma	0.93	0.43	1

## Discussion

To the best of the authors’ knowledge this is the widest study investigating the CEUS characteristics of malignant liver nodules in dogs as well as the first study comparing the CEUS features of different canine malignant liver lesions. The veterinary literature on this topic is limited and fragmentary; indeed there are just two studies systematically describing the CEUS features of canine liver nodules^6,8^, both of which consider only a limited number of cases. In fact, the study by O’Brien and others 2004^8^ is based on 32 cases in total (15 malignant and 17 benign) while the study by Nakamura and others (2010) is based on 33 dogs (6 normal, 16 with malignant nodules and 11 with benign lesions). Given the low number of cases, only benign and malignant lesions were statistically compared and the differences among different histopathological groups were not analysed.

In human medicine a vast literature^10^ describes the CEUS features of HCCs, which are reported to be mostly (97%) hyperenhancing during wash-in^11^ and with a slow wash-out^12^. Interestingly, although hyperenhancement during wash-in was the most common feature of the HCCs included in the present study (50/68–73.5%), there were remarkably higher percentages of cases showing no-enhancement (3/68–4.4%) or hypo/isoenhancement (15/68–22%). The intrinsic reason for such differences in the CEUS features of HCCs between humans and dogs is beyond the authors’ knowledge. We can, however, hypothesize that lesion dimension might play a role in such differences. Indeed, CEUS is often performed as part of the assessment of a human patient undergoing liver transplantation and, in such a scenario, strict cut-off values for lesion dimensions are used^13^. Liver transplantation is not performed on animal patients and therefore no cut-off values for liver lesion size have been chosen in the present study.

Rim-like hyperenhancement in the arterial phase followed by hypoenhancement in the portal and late phase are reported to be the typical CEUS features of ICC in human patients^10,12,14,15^. Interestingly, the canine ICCs included in the present study displayed a large variety of contrast- enhancement patterns and, although statistically significant differences between ICCs and HCCs were evident, no clear distinction between these two pathologies was possible based only on their CEUS appearance. Similarly to what is described for human patients^16^, the ICCs had a slower wash-out (and therefore a higher TTP) compared to HCCs.

Primary liver sarcomas are reported to be rare both in humans^17^ and in dogs^1^. The classification of the different types of sarcomas and the distinction between primary and metastatic liver sarcoma are challenging only by means of cytology. Indeed, histopathological examination is often needed to obtain a certain diagnosis. Therefore, in the present study, liver sarcomas have been considered as a single category. Angiosarcomas are reported to be the third most common liver malignancy in humans^18^, and these are described as being characterized by peripheral hyperenhancement during the arterial phase, followed by a rapid wash-out^15^. In this study, sarcomas displayed all the possible degrees of wash-in enhancement, with non-enhancing being the most common appearance. Further studies, possibly using histopathology as a reference standard are required to fully characterize the CEUS appearance of different sarcoma histotypes.

D’Onofrio *et al*. 2015 classifies liver metastases based on their CEUS features as hypovascular and hypervascular. Hypervascular metastases usually derive from sarcomas whereas hypovascular metastases usually derive from carcinomas^15^. Interestingly, the metastases from carcinomas included in this study displayed all the possible contrast-enhancement patterns, with the most common being hyperenhancement in the wash-in phase, followed by hypoenhancement in the wash-out phase. It is the authors’ opinion that an accurate comparison between the CEUS features of canine and human metastases is difficult mainly because histopathology rather than cytology could enable a more accurate discrimination of different histotypes of metastatic carcinomas.

The developed decision tree is, in our opinion, a very useful, and user-friendly tool for the veterinarians to predict the histotype of the hepatic lesions based on their CEUS features. The most interesting result is that only one lesion (the metastasis from cutaneous melanoma- later removed from the statistical analysis) was incorrectly classified as HCC. A limitation of this decision tree is that it starts from the assumption that the lesion is malignant because benign lesions were not included in the study.

The main limitation of the present study is that cytology was used as the reference standard to classify the samples. However, based on the different morphological features of the cells, it is possible to discriminate between ICC, HCC, sarcomas and metastatic carcinoma on the cytological examination alone^19–21^. Similar limitations apply to all the other veterinary studies describing the CEUS features of liver lesions in dogs^6,8,22^.

## Conclusions

According to the results of the present study some differences among the CEUS features of the different histotypes were evident. Nonetheless, as all the histotypes displayed all the possible patterns of contrast enhancement, a differentiation of liver masses in dogs based only on their CEUS features is not feasible and, therefore, cytology or histopathology is required.

## Methods

### Patients

Dogs referred to Ultravet (Ultravet, Via E. Fermi 59, San Giovanni in Persiceto, Bologna, Italy) and Tyrus Veterinary Clinic (Tyrus Veterinary Clinic, Via A. Bartocci 1/G, Terni, Italy) for specialty CEUS and cytopathological examination of previously ultrasonographically diagnosed liver masses were included. Patients referred between January 2010 and January 2019 were retrospectively selected by TB and SB; patients referred between February 2019 and August 2019 were prospectively selected by the same operators. Complete signalment and medical history were recorded for each patient.

Only dogs with a single liver mass evident on B-mode ultrasound examination, and with cytopathological diagnosis of malignancy, were included in the study. On the other hand, those patients (1) with cytopathological diagnosis of benign mass, (2) that underwent chemotherapy prior to the CEUS examination, (3) that had thrombosis of the hepatic vein, or congenital or acquired vascular abnormalities, were excluded from the study.

All the methods were carried out in accordance with relevant guidelines and regulations. This study was conducted according to the Italian law D. Leg.vo 26/2014 (that transposes the EU directive 2010/63/EU). As the data used in this study were part of the routine clinical activity no ethical committee approval was needed. Informed consent regarding the treatment of personal data was obtained from the owners.

### B-Mode examinations

Each patient underwent a B-mode ultrasonographic examination performed by two veterinarians (GR and PB with 18 and 15 years of experience in small animal ultrasonography, respectively) using three different ultrasonographic scanners: GE Logic E9 (GE Medical Systems), Esaote MyLab70 Gold (Esaote Italia) or Esaote Twice (Esaote Italia). Gain and time-gain compensation were appropriately adjusted during the examination. The qualitative features of individual lesions (both for the B-mode and the CEUS) were assigned following a consensus discussion; more specifically, after completion of the individual description, majority consensus between the two evaluators was used to assign the final features. The following qualitative features of the liver masses were collected during B-mode ultrasound: (1) echogenicity, compared to the surrounding liver parenchyma, classified as hypoechoic, hyperechoic, isoechoic or with mixed echogenicity; (2) aspect, classified as cystic or solid; (3) distribution, classified as focal or diffuse (when the mass involved an entire liver lobe or when the distinction between neoplastic and normal parenchyma was not clear).

### CEUS examinations

CEUS was performed by two veterinarians (GR and PB with 8 and 10 years of experience in liver CEUS respectively), using the following standardized protocol:

1. 8 hours fasting period prior to examination;
2. B-mode examination of the liver;
3. Sedation was performed in restless patients or if the lesion was located in a deep part of the liver or near the major vessels. In those cases, butorphanol tartrate was administered intramuscularly. This sedation protocol was chosen due to the reduced hemodynamic effects of butorphanol tartrate^23^;
4. A 21 G intravenous cannula placed in the cephalic vein (saline solution was administered during the entire procedure);
5. Sonovue® (Bracco Imaging BV, Geneva, Switzerland) was administered intravenously at the dose of 0.05 ml/kg, followed by a 5 ml of saline flush through a 3way stopcock with an extension tube (containing 0.5 ml of fluid) directly connected to the intravenous cannula;
6. Each lesion was scanned continuously for at least one minute, or until the end of the wash-out phase. The mechanical index was set to a low value (0.02). Each scan was entirely recorded.

All the CEUS examinations were reviewed by the same veterinarians (GR and TB). The following qualitative contrast-enhancement features of the lesions were evaluated during wash-in: (1) enhancement degree (hyperenhancing, hypoenhancing, isoenhancing or non-enhancing) compared to the surrounding liver parenchyma; (2) homogeneity (homogeneous or inhomogeneous); (3) distribution of the contrast agent (central, peripheral or diffuse). Similarly, during the wash-out phase the following qualitative features of the lesions were evaluated: (1) enhancement degree compared to the surrounding liver parenchyma (lesions that were hyperenhancing during the wash-in phase and isoenhancing during the wash-out, and lesions that were isoenhancing or hypoenhancing during both wash-in and wash-out were classified as having “no wash-out”; lesions that were hyperenhancing or isoenhancing during wash-in phase and hypoenhancing during wash-out phase were classified as having hypoenhancing wash-out); (2) homogeneity (homogeneous or inhomogeneous); (3) pattern of contrast-medium decrease (central, peripheral or diffuse). The lesion margins were evaluated during all the CEUS examinations and were classified as: (1) “clear and regular”; (2) “clear and irregular”; (3) “unclear and regular”; (4) “unclear and irregular”.

The original Digital Imaging and Communication in Medicine (DICOM) files were no longer available and, therefore, all the examinations were stored as AVI files. A MATLAB script was developed by one of the authors (TB) to calculate the time-intensity curves from the videos. The MATLAB script extracted all the frames from the video and saved them as images in a folder. Thereafter, two regions of interest (ROI) were manually placed by one operator (SB) on: (1) a contrast enhancing portion of the the liver mass (if the lesion was enhacing) 2) a portion of ultrasonographically normal liver tissue. The mean grey value of the ROI was then calculated for all the frames and plotted to create a time-intensity curve. Based on the time-intensity curves, the following quantitative parameters were calculated on the liver mass: (1) time to enhancement (TTE), as the time from the injection to the beginning of the signal from the lesion tissue; (2) time to peak (TTP), as the maximum intensity of lesion enhancement measured from injection of the contrast medium; (3) time to wash-in (TTWI), as the difference between TTP and TTE.

### Cytopathological examinations

Liver masses were sampled through US-guided fine needle aspiration (FNA). The cytological slides were air-dried and stained with May-Grünwald-Giemsa stain. The cytological examinations were performed by trained pathologists hired specifically for this service. HCCs were diagnosed when the hepatocytes had marked criteria of atypia^20^ or, if the hepatocytes were relatively normal-appearing, when they were dissociated and not in clusters, when they were arranged in acinar or palisading formations, or when there were naked nuclei and capillaries^24^. ICCs were diagnosed when there were densely packed epithelial cells, arranged in sheets or tubular formations, with moderate-to-marked criteria of atypia^20^. Sarcomas were diagnosed when the cells were spindle-shapes, with moderate-to-marked criteria of atypia^20^. Metastatic carcinomas were diagnosed when the epithelial cells did not resemble hepatocytes or bile duct cells and had marked criteria of atypia or were anaplastic.

### Statistics and data analysis

The statistical analysis has been performed on the results of B-mode and CEUS ultrasounds of the retrospective cases. The cases collected during the prospective phase of the study have been classified based on the decision tree formulated on the statistics results.

Patients were classified into four groups, based on the results of the cytological analysis as HCC, ICC, sarcoma or metastasis.

All the statistical analyses were performed with the R programming language (R Core Team, 2013). R: a language and environment for statistical computing. R Foundation for Statistical Computing, Vienna, Austria, URL http://www.R-project.org/). Differences in the distribution frequency of the qualitative features of B-mode and CEUS between the four groups were evaluated using the Chi-squared test^25^ or the Fisher exact method. A Shapiro-Wilk test was used to check normal distribution of continuous variables. Quantitative features were compared using the one-way ANOVA test for normally distributed data, and the Kruskal-Wallis test for non-normally distributed data. Post-hoc tests, using the t-test with Bonferroni correction for normally distributed data and the pairwise comparison with a Mann and Whitney test for non-normally distributed data, were performed. The results were considered statistically significant with a P-value less than 0.05.

A decision tree was developed, based on the findings of the retrospective cases, using the rpart.plot package of the R programming language. Thereafter, the prospectively collected cases have been classified based on the decision tree. Overall accuracy, balanced accuracy, precision, and recall have been calculated using the mltest package for the R programming language.

## Supplementary information

Supplementary information 1.

Supplementary information 2.
