# Supplementary material for: Contrast-enhanced ultrasound features of malignant focal liver masses in dogs
Source: Sci Rep. 2020 Apr 8;10:6076. doi: 10.1038/s41598-020-63220-3 (PMC7142119; doi:10.1038/s41598-020-63220-3)
Supplement: Supplementary file 1 — Supplementary information 1. [file 41598_2020_63220_MOESM1_ESM.docx]

# Contrast-enhanced ultrasound features of malignant focal liver masses in dogs

Silvia Burti^1^, Alessandro Zotti^1^, Giuseppe Rubini^2^, Riccardo Orlandi^3^, Paolo Bargellini^3^, Federico Bonsembiante^1,4^, Tommaso Banzato^1*^.

*^1^Department of Animal Medicine, Productions and Health, University of Padua, Viale dell’Università 16, Legnaro, Italy.*

*^2^ ULTRAVET, Via E. Fermi 59, San Giovanni in Persiceto, Bologna, Italy.*

*^3^ Tyrus Veterinary Clinic, Via A. Bartocci 1/G, Terni, Italy.*

*^4^ Department of Comparative Biomedicine and Food Science, University of Padua, Viale dell’Università 16, Legnaro, Italy.*

Complete qualitative and quantitative analysis of the test cases

|  | **B-Mode** | | | **CEUS** | | | | | | | | | |  |
| --- | --- | --- | --- | --- | --- | --- | --- | --- | --- | --- | --- | --- | --- | --- |
|  |  |  |  | **Wash-In** | | | **Wash-Out** | | |  | **Quantitative features** | | |  |
| **Cytological diagnosis** | **Echogenicity** | **Aspect** | **Diffusion** | **Enhancement** | **Homogeneity** | **Distribution** | **Enhancement** | **Homogeneity** | **Pattern** | **Margins** | **TTE** | **TTP** | **TTWI** | **Decision Tree** |
| HCC | Hypoechoic | Solid | Focal | Hyperenhancing | Inhomogeneous | Peripheral | No wash out | - | - | Unclear Irregular | 5 | 13 | 8 | HCC |
| HCC | Mixed | Cystic | Focal | Hyperenhancing | Inhomogeneous | Diffuse | Hypoenhancing | Inhomogeneous | Diffuse | Unclear Irregular | 7 | 23 | 16 | HCC |
| HCC | Hypoechoic | Solid | Diffuse | Hypoenhancing | Inhomogeneous | Central | No wash out | - | - | Unclear Irregular | 8 | 25 | 17 | ICC |
| HCC | Hyperechoic | Cystic | Focal | Hyperenhancing | Inhomogeneous | Diffuse | No wash out | - | - | Unclear Irregular | 7 | 14 | 7 | HCC |
| HCC | Hyperechoic | Solid | Diffuse | Hyperenhancing | Inhomogeneous | Diffuse | No wash out | - | - | Unclear Irregular | 9 | 18 | 9 | HCC |
| HCC | Mixed | Solid | Focal | Hyperenhancing | Inhomogeneous | Diffuse | No wash out | - | - | Clear Irregular | 7 | 13 | 6 | HCC |
| HCC | Hyperechoic | Solid | Focal | Hypoenhancing | Inhomogeneous | Diffuse | Hypoenhancing | Homogeneous | Diffuse | Unclear Irregular | 10 | 23 | 13 | ICC |
| HCC | Mixed | Cystic | Diffuse | Hyperenhancing | Homogeneous | Peripheral | No wash out | - | - | Unclear Regular | 4 | 15 | 11 | Sarcoma |
| HCC | Hyperechoic | Solid | Diffuse | Hypoenhancing | Inhomogeneous | Diffuse | No wash out | - | - | Unclear Irregular | 9 | 28 | 17 | ICC |
| HCC | Hyperechoic | Solid | Focal | Isoenhancing | Homogeneous | Diffuse | No wash out | - | - | Unclear Irregular | 5 | 12 | 7 | HCC |
| HCC | Hyperechoic | Solid | Focal | Hyperenhancing | Homogeneous | Peripheral | Hypoenhancing | Inhomogeneous | Centrifugal | Clear Irregular | 5 | 13 | 8 | HCC |
| HCC | Hyperechoic | Solid | Diffuse | Hyperenhancing | Inhomogeneous | Diffuse | Hypoenhancing | Inhomogeneous | Diffuse | Unclear Irregular | 5 | 16 | 11 | HCC |
| HCC | Hyperechoic | Solid | Focal | Hyperenhancing | Inhomogeneous | Central | No wash out | - | - | Clear Irregular | 6 | 12 | 6 | HCC |
| HCC | Hypoechoic | Solid | Focal | Hyperenhancing | Homogeneous | Diffuse | No wash out | - | - | Clear Irregular | 5 | 13 | 8 | HCC |
| HCC | Mixed | Solid | Diffuse | Hyperenhancing | Homogeneous | Diffuse | Hypoenhancing | Inhomogeneous | Diffuse | Clear Irregular | 15 | 23 | 8 | HCC |
| HCC | Mixed | Solid | Diffuse | Hyperenhancing | Inhomogeneous | Diffuse | Hypoenhancing | Inhomogeneous | Centrifugal | Clear Irregular | 5 | 10 | 5 | HCC |
| HCC | Mixed | Cystic | Diffuse | Hyperenhancing | Inhomogeneous | Diffuse | Hypoenhancing | Homogeneous | Diffuse | Clear Irregular | 5 | 14 | 9 | HCC |
| HCC | Hypoechoic | Solid | Focal | Hyperenhancing | Inhomogeneous | Diffuse | No wash out | - | - | Clear Irregular | 8 | 23 | 15 | HCC |
| HCC | Mixed | Solid | Diffuse | Hyperenhancing | Inhomogeneous | Diffuse | No wash out | - | - | Clear Irregular | 12 | 20 | 8 | HCC |
| HCC | Hyperechoic | Cystic | Diffuse | Hyperenhancing | Inhomogeneous | Diffuse | Hypoenhancing | Inhomogeneous | Diffuse | Unclear Irregular | 9 | 23 | 14 | HCC |
| HCC | Hyperechoic | Solid | Focal | Hyperenhancing | Inhomogeneous | Diffuse | No wash out | - | - | Clear Irregular | 6 | 10 | 4 | HCC |
| HCC | Mixed | Solid | Focal | Isoenhancing | Homogeneous | Diffuse | No wash out | - | - | Unclear Irregular | 10 | 26 | 16 | HCC |
| HCC | Hyperechoic | Solid | Focal | Hyperenhancing | Homogeneous | Diffuse | No wash out | - | - | Clear Irregular | 8 | 17 | 9 | HCC |
| HCC | Hypoechoic | Solid | Focal | Hyperenhancing | Homogeneous | Diffuse | No wash out | - | - | Clear Irregular | 9 | 13 | 4 | HCC |
| HCC | Hypoechoic | Solid | Focal | Hyperenhancing | Homogeneous | Diffuse | Hypoenhancing | Inhomogeneous | Diffuse | Clear Irregular | 11 | 16 | 5 | HCC |
| HCC | Hypoechoic | Solid | Focal | Hyperenhancing | Inhomogeneous | Diffuse | No wash out | - | - | Unclear Irregular | 5 | 17 | 12 | HCC |
| HCC | Mixed | Solid | Diffuse | - | - | - | - | - | - | Clear Irregular | - | - | - | Sarcoma |
| HCC | Hypoechoic | Solid | Diffuse | - | - | - | - | - | - | Clear Irregular | - | - | - | Sarcoma |
| ICC | Mixed | Solid | Diffuse | Hypoenhancing | Inhomogeneous | Diffuse | No wash out | - | - | Unclear Irregular | 14 | 29 | 15 | ICC |
| ICC | Hypoechoic | Solid | Focal | - | - | - | - | - | - | Clear Irregular | - | - | - | Sarcoma |
| ICC | Mixed | Cystic | Diffuse | Hypoenhancing | Inhomogeneous | Diffuse | No wash out | - | - | Unclear Irregular | 7 | 18 | 11 | ICC |
| Sarcoma | Mixed | Cystic | Diffuse | Hyperenhancing | Inhomogeneous | Peripheral | No wash out | - | - | Unclear Irregular | 13 | 24 | 11 | Sarcoma |
| Sarcoma | Hyperechoic | Cystic | Focal | Hypoenhancing | Homogeneous | Peripheral | No wash out | - | - | Clear Irregular | 9 | 20 | 11 | Sarcoma |
| Sarcoma | Hyperechoic | Cystic | Focal | - | - | - | - | - | - | Clear Irregular | - | - | - | Sarcoma |
| Mts | Mixed | Cystic | Diffuse | Hyperenhancing | Homogeneous | Diffuse | Hypoenhancing | Homogeneous | Diffuse | Clear Irregular | 8 | 12 | 4 | HCC |
