# Supplementary material for: Contrast-enhanced ultrasound features of malignant focal liver masses in dogs
Source: Sci Rep. 2020 Apr 8;10:6076. doi: 10.1038/s41598-020-63220-3 (PMC7142119; doi:10.1038/s41598-020-63220-3)
Supplement: Supplementary file 2 — Supplementary information 2. [file 41598_2020_63220_MOESM2_ESM.pdf]

# Contrast-enhanced ultrasound features of malignant focal liver masses in dogs

Silvia Burti[1], Alessandro Zotti[1], Giuseppe Rubini[2], Riccardo Orlandi[3], Paolo Bargellini[3], Federico Bonsembiante[1,4], Tommaso Banzato[1*].

[1]*Department of Animal Medicine, Productions and Health, University of Padua, Viale dell'Università 16, Legnaro, Italy.*

[2] *ULTRAVET, Via E. Fermi 59, San Giovanni in Persiceto, Bologna, Italy.*

[3] *Tyrus Veterinary Clinic, Via A. Bartocci 1/G, Terni, Italy.*

[4] *Department of Comparative Biomedicine and Food Science, University of Padua, Viale dell'Università 16, Legnaro, Italy.*

**Example of time-intensity curves showing how the quantitative features of time to enhancement (TTE), time to peak (TTP) and time to wash-in (TTWI) were calculated.**

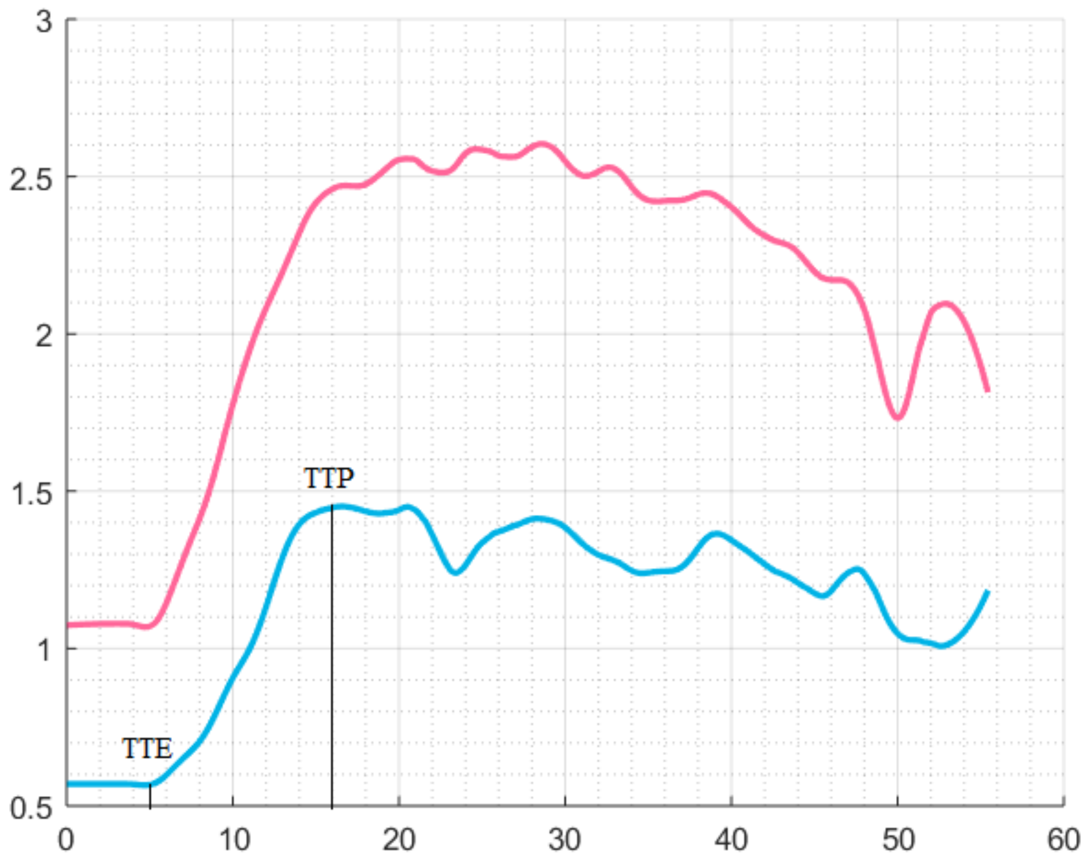


Time-intensity curve of a bile duct carcinoma, showing hypoenhancement, compared to the ultrasonographically normal liver parenchyma, during all the phases of the study. The light-blue curves refers to the mass, the red curves refers to the liver parenchyma. Sarcoma, having not-enhancement; d) Metastasis, showing hypoenhancement during all the examination.

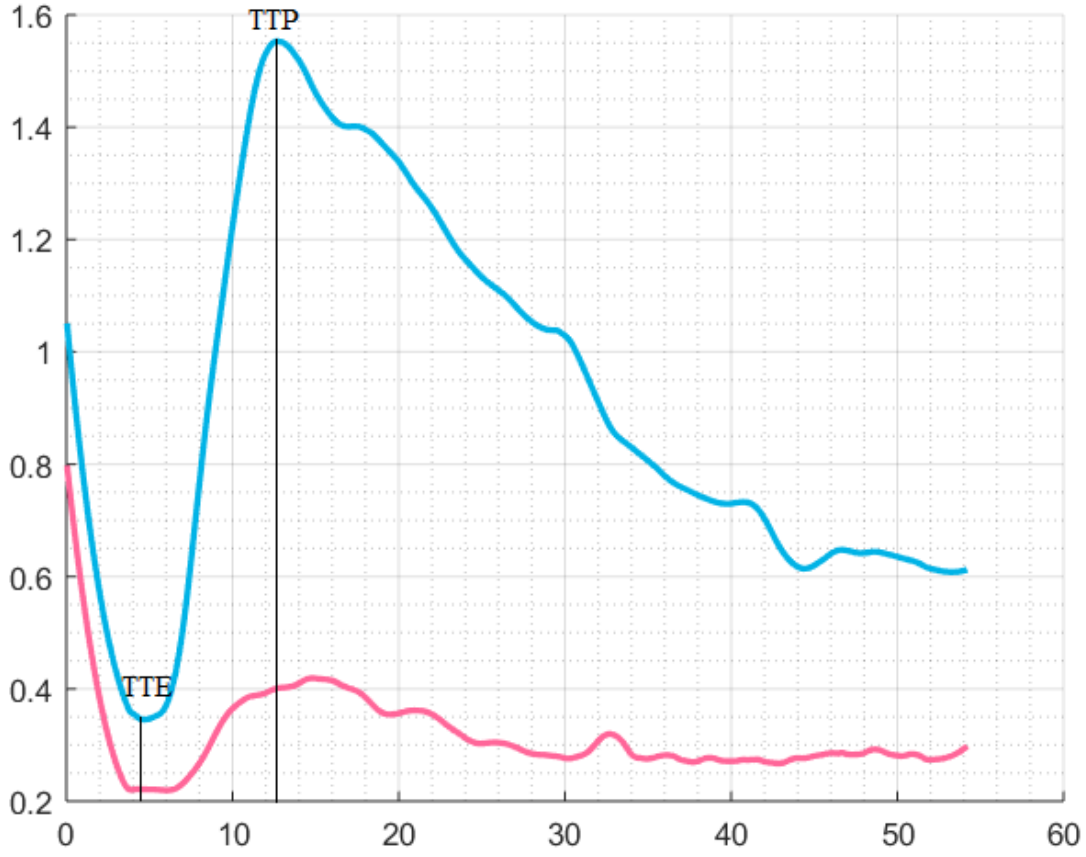


Time-intensity curve of a hepatocellular carcinoma, showing hyperenhancement, compared to the ultrasonographically normal liver parenchyma, during all the phases of the study. The light-blue curves refers to the lesion, the red curves refers to the liver parenchyma.

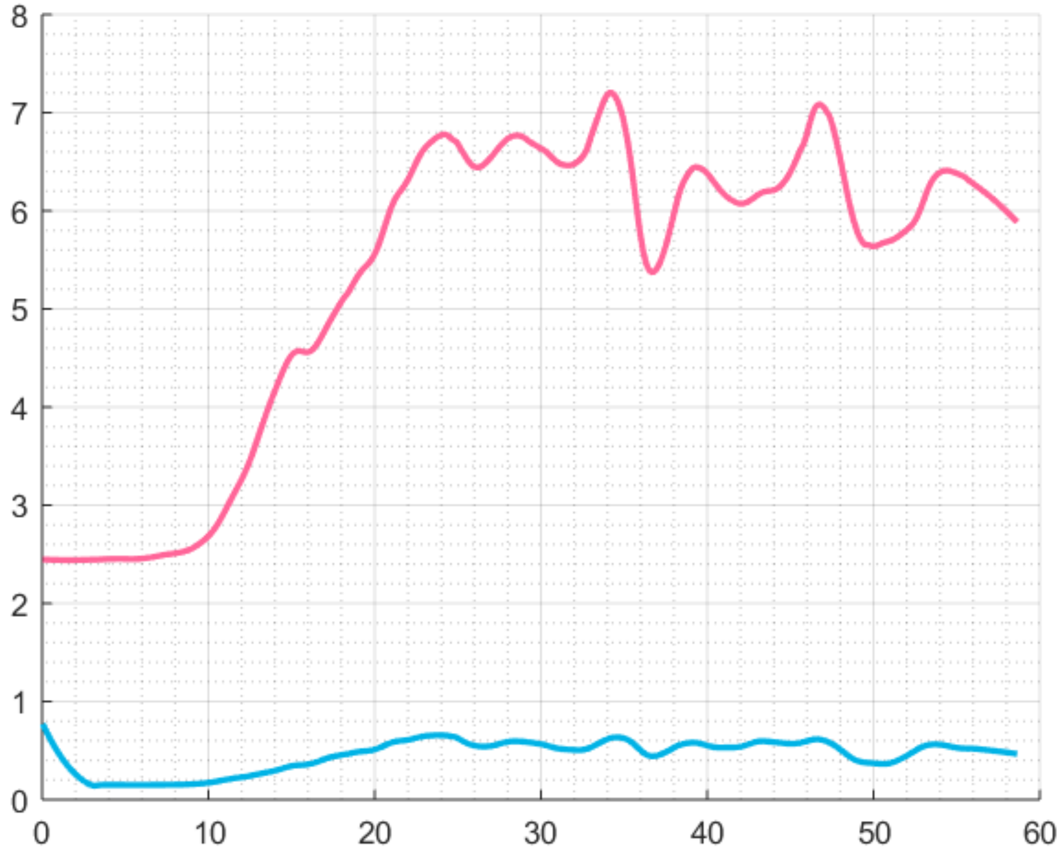


Time-intensity curve of a not-enhancing sarcoma. The light-blue curves refers to the mass, the red curves refers to the liver parenchyma.

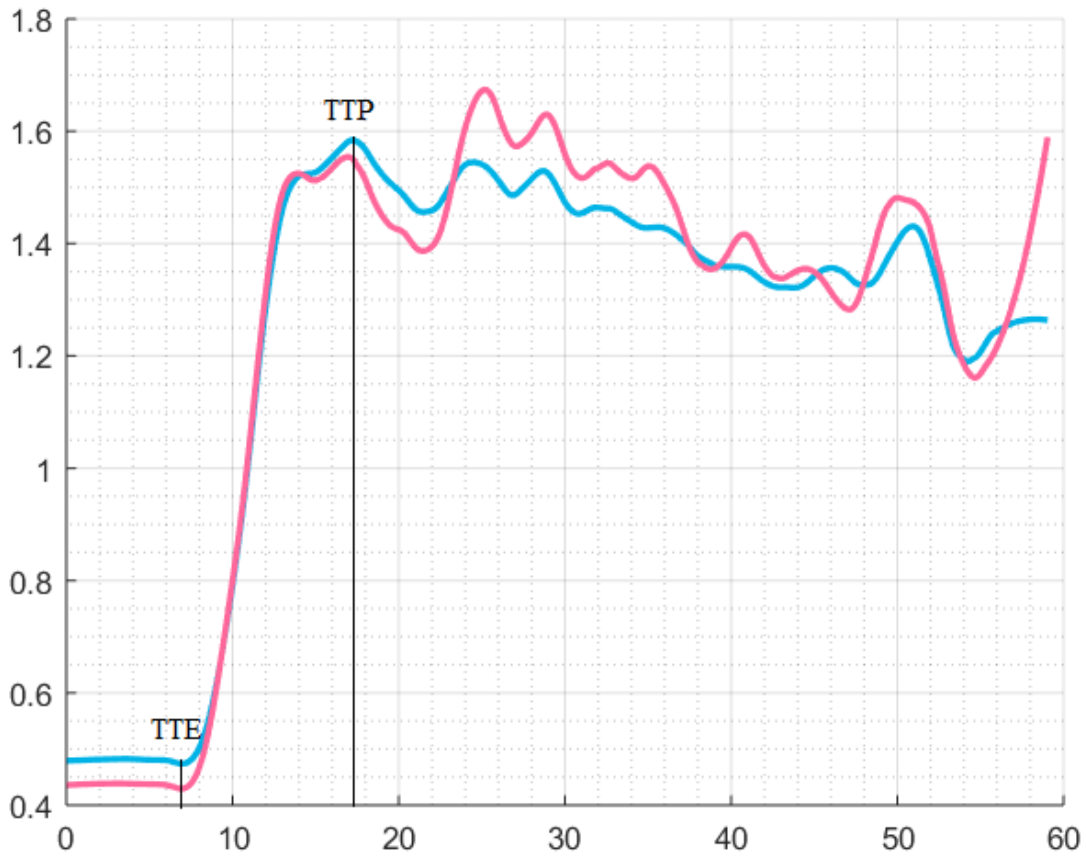


Time-intensity curve of a metastasis showing hyperenhancement followed by hypoenhancement. The light-blue curves refers to the mass, the red curves refers to the liver parenchyma.
